# Motivations and Preferences for Self-Sampled Human Papillomavirus Testing Among Average—and High-Risk Patients: An Exploratory Analysis

**DOI:** 10.1089/whr.2024.0180

**Published:** 2025-03-28

**Authors:** Rebecca Morgis, Ashley Wong, Lisa M. Witmer, Anne Kantner, Megan Mendez-Miller, Sarah I. Ramirez, Mack T. Ruffin, Jennifer L. Moss

**Affiliations:** Penn State College of Medicine, Hershey, Pennsylvania, USA.

**Keywords:** cervical cancer, cancer screening, human papillomavirus (HPV), self-sample

## Abstract

**Objectives:**

Cervical cancer screening rates fall below national goals. At-home self-sampled tests for human papillomavirus (HPV) may improve screening rates. This study assesses the acceptability of self-sampled HPV testing with respect to motivating factors and preference among average-risk patients (undergoing routine screening) and high-risk patients (receiving follow-up care after abnormal screening results).

**Materials and Methods:**

This cross-sectional study sample consisted of 46 participants (female, ages 30–65), including average-risk (n = 35) and high-risk (n = 11) patients, who had already received clinician-collected cervical cancer screening. Participants completed a self-sampled HPV test and a survey. Motivators included cervical cancer screening facilitators, sexual history, health care factors, and feelings during self-sampled test. We analyzed the relationships between these constructs and test modality preference for their next cervical cancer screening (i.e., self-sampled HPV testing at home vs. other preference).

**Results:**

Few participants experienced negative feelings during self-sampled HPV testing (uncomfortable: 20%; anxious: 22%; and unpleasant: 15%). Overall, 57% of participants would prefer to complete a self-sampled HPV test at home for their next cervical cancer screening compared with other test options. Test modality preference for their next cervical cancer screening did not differ for average- versus high-risk patients, and it did not vary by any of the motivating factors we assessed (all *p* > 0.05).

**Conclusions:**

Acceptability of self-sampled HPV testing at home is high, with little difference in attitudes observed across patient characteristics. These findings demonstrate that self-sampled HPV testing may be an effective tool for increasing cervical cancer screening, even among high-risk patients who have previously had abnormal screening results.

## Introduction

The burden of cervical cancer persists in the United States, with an incidence rate of 7.9 cases per 100,000 females per year and a mortality rate of 2.2 deaths per 100,000 females per year.^1^ Mortality has decreased substantially in the last decades,^1^ with the widespread implementation of cervical cancer screenings. Screening measures include cervical cytology, high-risk human papillomavirus (HPV) testing, or combination screening.^2^ If a patient has abnormal results on their routine cervical cancer screening, risk stratification guidelines determine the next steps for diagnostic resolution, for example, additional testing or shorter interval follow-up screening.^3^ However, receipt of cervical cancer screening is suboptimal, with only 80.5% of women ages 21–65 up-to-date with screening, which falls short of Healthy People 2030 goals.^4^

Potential barriers to accessing cervical cancer screening include time constraints, lack of awareness, and prior negative screening experiences (e.g., discomfort and embarrassment).^5,6^ Self-sampling for HPV testing serves as a possible intervention to increase cervical cancer screening.^7,8^ Self-sampling allows individuals to collect their own cervical swab sample and provide it to a lab for testing, and it does not require a speculum exam to be collected. A rich literature supports the accuracy^9–14^ and acceptability^9,10,15–17^ of self-sampled compared with clinician-collected HPV tests. However, data are lacking on the acceptability of self-sampled HPV testing among patients with diverse screening histories.

This pilot study examined the acceptability of self-sampled HPV testing among average- and high-risk patients. Average-risk patients were individuals receiving routine cervical cancer screenings, and high-risk patients were those with previous abnormal screening results that led to further follow-up (e.g., colposcopy). Participants completed both clinician-collected and self-collected HPV testing, and we examined their motivating factors and preferences for screening modality as key measures of acceptability of self-sampled HPV testing.

## Materials and Methods

### Study participants and procedures

This study was conducted at a suburban health system located in central Pennsylvania.^18,19^ We partnered with several health care providers in the family medicine and gynecology departments who routinely perform cervical cancer screening and diagnostic procedures. Partnering providers were involved in participant recruitment and received weekly reminder messages about study progress.

#### Participant identification and enrollment

Study inclusion criteria included being a patient in the health system, ages 30–65 years, female sex, intact cervix, and English proficiency. Exclusion criteria included current pregnancy, complete hysterectomy, history of cervical treatment for abnormal cytology/HPV test (i.e., cryotherapy and loop electrosurgical excision procedure), cognitive impairment, or current incarceration. “Average-risk” participants were patients who received routine cervical cancer screening completed by a clinician.^18^ “High-risk” participants were patients in the family medicine or gynecology departments with previously established abnormal results on cytology and/or HPV testing that required colposcopy for further assessment.^19^ Notably, these eligibility criteria ensured that all participants would have a recently completed cervical cancer screening (with a clinician-collected sample) documented in their electronic medical record. In clinical practice, high-risk patients would likely not undertake self-sampled HPV testing as a screening approach; instead, in this study, we enrolled high-risk patients to ensure that we have an adequate sample of patients with abnormal HPV test results to facilitate statistical analysis.

Between December 2020 and October 2022, participants were recruited through patient–provider conversations, provider referral to the study team, or patient outreach via mailed invitations. A study team member contacted patients to invite them to participate, confirm study eligibility, and obtain verbal informed consent for participation. All patients received normal clinical care from their clinicians (e.g., gynecological exam, cytology, HPV test, and colposcopy), regardless of study participation.

#### Study procedures

After enrollment, participants received via mail a printed copy of the informed consent form, a kit for self-sampled HPV testing, and instructions for use. Participants also received materials (i.e., processing form and prepaid return envelope) for returning the completed test kit to the testing laboratory, as well as a thank-you letter from the principal investigator. Participants were asked to complete and return the self-sampled HPV test kit within a week of receiving it. If the test kit was not returned to the lab within 2 weeks, a study team member called to check if participants had collected their sample or had any questions. After returning their test kit, participants completed a brief survey focused on the acceptability of self-sampled HPV testing. The primary study outcomes, described elsewhere, examined concordance between the clinician-collected and the self-sampled HPV test results,^14^ whereas the current analysis examined acceptability of the self-sampled HPV tests.

### Measures

Study measures included demographic characteristics, motivating factors for screening, and preference of HPV testing modality, which were drawn from the participant surveys.

#### Demographics

Demographic characteristics included age-group, race/ethnicity, educational attainment, annual household income, and marital status.

#### Motivating factors for cervical cancer screening and self-sampled HPV testing

First, we assessed cervical cancer screening facilitators. Items asked the extent to which participants agreed or disagreed that they need to get checked for cervical cancer and that they had enough time to get checked. Response options are categorized as strongly/somewhat agree versus strongly/somewhat disagree.

Second, we assessed the number of lifetime sexual partners, which could impact cervical cancer screening behaviors. Based on the distribution of responses, we categorized lifetime number of sexual partners into 1–3 partners, 4–7 partners, or 8+ partners. Third, we assessed health care factors, *that is*, lifetime cancer history and receipt of ≥1 dose of HPV vaccine.

Fourth, we assessed participants’ feelings while completing the self-sampled HPV test, including feeling uncomfortable, feeling anxious, and finding the test unpleasant. Conservatively, response options are categorized as not at all versus somewhat/very.

We assessed a number of other potential motivating factors for cervical cancer screening and self-sampled HPV testing. Participants were almost unanimous in their responses to these items (e.g., 98% strongly/somewhat agreed that they know how to get screened; 91% had attended a preventive check-up in the last year; 100% felt “not at all” embarrassed when completing the self-sampled HPV test). The limited variability in responses to these items precluded their statistical analysis. See Supplementary Table S1 for complete details.

#### Preference of cervical cancer screening modality

Our key outcome measure was preferred cervical cancer screening modality. Participants read the question, “Thinking about the HPV test you completed at home and a Pap and HPV test you received at the doctor’s office, which one would you want the next time you need to get checked for cervical cancer?” and responded with (1) HPV test at home, (2) Pap test at doctor’s office, (3) HPV test at doctor’s office, (4) No preference, or (5) Don’t know. We categorized responses as (1) preferring an HPV test at home versus (2) any other preference.

### Statistical analysis

We generated descriptive statistics for the demographic characteristics of the study sample. We used Fisher’s exact tests to assess differences in these variables for average-compared with high-risk patient cohorts. Because this analysis determined that cohorts differed by age group and marital status (Table 1), subsequent analyses controlled for these variables. Next, we summarized participants’ responses to items assessing motivating factors related to screening.

**Table 1.** Demographic Characteristics of Study Participants (Ages 30–65 Years, Female Sex) Completing Clinician-Collected and Self-Sampled for Human Papillomavirus Testing and Comparisons Between Average-Risk and High-Risk Patient Cohorts

	Total (*n* = 46)		Average-risk cohort^a^ (*n* = 35)		High-risk cohort^b^ (*n* = 11)	
Age-group						
30–49 years	25	54%	15	43%	10	91%
50–65 years	21	46%	20	57%	1	9%
Race/ethnicity						
Non-Hispanic White	38	83%	31	89%	7	64%
Hispanic	5	11%	2	6%	3	27%
Other	3	7%	2	6%	1	9%
Educational attainment						
Less than a 4-year college degree	13	28%	8	23%	5	45%
A 4-year college degree or higher	33	72%	27	77%	6	55%
Annual household income						
Less than $50,000	7	16%	5	15%	2	18%
$50,000 or more	37	84%	28	85%	9	82%
Marital status						
Not married	14	30%	8	23%	6	55%
Married/living as married	32	70%	27	77%	5	45%

^a^ Participants in the average-risk cohort were patients receiving routine cervical cancer screenings.

^b^ Participants in the high-risk cohort were patients with previous abnormal screening results that led to further follow-up (e.g., colposcopy).

Finally, we used logistic regression models to evaluate the relationships between motivating factors and preference for cervical cancer screening modality, adjusting for age group and marital status. We used odds ratios and 95% confidence intervals to summarize the strength of the relationships between each motivating factor and preference for self-sampled HPV testing compared with other cervical cancer screening modalities.

All analyses were conducted in SAS version 9.4 (Cary, NC) using a two-tailed *p*-value of 0.05. The Pennsylvania State University Human Research Protection Program approved data collection and analysis for this project (study #13464 and #14033). All participants provided verbal informed consent at enrollment.

## Results

### Participant demographics

In total, 47 participants completed the study. One participant did not provide their preference for cervical cancer screening modality (our key outcome variable), so we excluded their data, resulting in an analytic sample of 46, including 35 participants in the average-risk cohort and 11 participants in the high-risk cohort (Table 1). Most participants were non-Hispanic White (83%), had at least a college degree (72%), and had an annual household income of $50,000 or more (84%). Compared with participants in the average-risk cohort, participants in the high-risk cohort were more likely than participants in the average-risk cohort to be in the younger age group (*p* < 0.01) and to be not married (*p* = 0.05).

### Motivating factors for cervical cancer screening and self-sampled HPV testing

Most participants agreed that they needed (74%) and had adequate time (93%) to accomplish screening (Table 2). The lifetime number of sexual partners was 1–3 partners for 36% of participants, 4–7 partners for 25%, and 8+ partners for 39%. Most participants did not have a history of cancer (91%) or HPV vaccination (82%). Less than a quarter of participants reported that self-sampled HPV testing made them somewhat or very uncomfortable (20%) or anxious (22%), and 15% found the test unpleasant (15%). Although HPV vaccination was more common among participants in the high-risk group (55%) than the average-risk group (6%) (*p* < 0.01), the remaining factors did not differ across risk groups (all *p* > 0.09).

**Table 2.** Motivating Factors for Cervical Cancer Screening and Self-Sampled HPV Testing Among Study Participants (n = 46, Ages 30–65 Years, Female Sex)

	*n*	%
Cervical cancer screening facilitators		
Need to get checked		
Strongly/somewhat disagree	12	26%
Strongly/somewhat agree	34	74%
Have enough time to get checked		
Strongly/somewhat disagree	3	7%
Strongly/somewhat agree	43	93%
Sexual history		
Lifetime number of sexual partners		
1–3 partners	16	36%
4–7 partners	11	25%
8+ partners	17	39%
Health care factors		
History of cancer		
No	42	91%
Yes	4	9%
History of HPV vaccination		
No	37	82%
Yes	8	18%
Feelings during self-sampled test		
Feel uncomfortable		
Not at all	37	80%
Somewhat/very	9	20%
Feel anxious		
Not at all	36	78%
Somewhat/very	10	22%
Find the test unpleasant		
Not at all	39	85%
Somewhat/very	7	15%

HPV, human papillomavirus.

### Preference of cervical cancer screening modality

When thinking about their next cervical cancer screening, 57% of participants would want to complete a self-sampled HPV test at home, whereas 43% indicated other preferences (17% had no preference and 26% preferred a Pap or HPV test at the doctor’s office; Table 3). Controlling for age group and marital status, preference for self-sampled HPV testing did not differ across risk group or any of the motivating factors that we assessed (all *p* > 0.05).

**Table 3.** Relationships Between Motivating Factors for Cervical Cancer Screening and Self-Sampled HPV Testing Among Study Participants (n = 46, Ages 30–65 Years, Female Sex)

	Other preference^a^		HPV test at home			
	*n*	%	*n*	%	OR	95% CI
Overall	20	43%	26	57%		
Risk group						
Average-risk cohort	5	45%	6	55%	(ref)	
High-risk cohort	15	43%	20	57%	0.90	(0.23–3.52)
Cervical cancer screening facilitators						
Need to get checked						
Strongly/somewhat disagree	5	42%	7	58%	(ref)	
Strongly/somewhat agree	15	44%	19	56%	0.91	(0.24–3.50)
Have enough time to get checked						
Strongly/somewhat disagree	1	33%	2	67%	(ref)	
Strongly/somewhat agree	19	44%	24	56%	0.63	(0.05–7.48)
Sexual history						
Lifetime number of sexual partners						
1–3 partners	7	44%	9	56%	(ref)	
4–7 partners	6	55%	5	45%	0.66	(0.14–3.14)
8+ partners	7	41%	10	59%	1.27	(0.28–5.73)
Health care factors						
History of cancer						
No	18	43%	24	57%	(ref)	
Yes	2	50%	2	50%	0.75	(0.10–5.85)
History of HPV vaccination						
No	17	46%	20	54%	(ref)	
Yes	3	38%	5	63%	1.44	(0.28–7.43)
Feelings during self-sampled test						
Feel uncomfortable						
Not at all	16	43%	21	57%	(ref)	
Somewhat/very	4	44%	5	56%	0.95	(0.22–4.16)
Feel anxious						
Not at all	15	42%	21	58%	(ref)	
Somewhat/very	5	50%	5	50%	0.71	(0.17–2.91)
Find the test unpleasant						
Not at all	16	41%	23	59%	(ref)	
Somewhat/very	4	57%	3	43%	0.49	(0.09–2.71)

^a^ Includes response options of preferences for “Pap test at doctor’s office,” “HPV test at doctor’s office,” “No preference,” and “Don’t know.”

CI, confidence interval; OR, odds ratio; ref, reference category.

## Discussion

The findings from our study demonstrate high acceptability of self-sampled HPV testing. Participants rarely reported experiencing negative feelings (e.g., anxiety) during the self-sampling process. Furthermore, the majority of participants indicated a preference for self-sampled HPV testing over alternative cervical cancer screening options. These outcomes were fairly uniform across patient subgroups, including for average- versus high-risk patients (i.e., those attending routine screening and those who had already received abnormal screening results, respectively) and across other predictors of cervical cancer screening behaviors. These findings add to the research literature by demonstrating similarity in motivations and preferences for self-sampled HPV testing across patients with diverse risk profiles and screening histories.

One of the main goals of this study was to compare motivations and preferences for self-sampled HPV testing for average-risk patients compared with high-risk patients. High-risk patients were those with previous abnormal results on (clinician-collected) cervical cancer screening, and who had been referred for colposcopy. Patients who receive abnormal cervical cancer screening results often experience elevated confusion and anxiety,^20–22^ which could hinder acceptability of a novel HPV testing modality among these patients. However, outcomes, including preference for self-sampled HPV testing for the next cervical cancer screening, were equivalent across patient risk groups. This finding suggests that self-sampled HPV testing could serve as an effective tool for increasing cervical cancer screening, even among high-risk and vulnerable patients. As noted above, the primary outcomes for this research project focused on concordance between the clinician-collected and the self-sampled HPV test results.^14^ In that study, we reported high concordance in the average-risk group and moderate concordance in the high-risk group, whereas the current analysis examined the acceptability of the self-sampled HPV tests. Continuing efforts to maximize accuracy across screening modalities are necessary to ensure acceptability and relevance of these tools to patients.

In the present study, we did not find any associations between the assessed motivating factors and testing modality preference for the next time participants need to receive cervical cancer screening. That is, preference for self-sampled HPV testing did not vary by previously identified cervical cancer screening facilitators, sexual history, health care factors, or feelings while completing the self-sampled test. This finding suggests that the acceptability of self-sampled HPV testing is fairly uniform across intrapersonal factors that have previously been associated with cervical cancer screening. In the future, programs aiming to increase cervical cancer screening through outreach with self-sampled HPV tests could use a population-wide approach; this population-wide approach could be more efficient than tailored approaches that adjust intervention components based on intrapersonal factors.^23^ That said, the greatest absolute differences in test modality preference were for having enough time to get screened and HPV vaccination history. Future studies should explore whether these constructs predict acceptability and screening behaviors with self-sampled HPV testing in larger samples.

Our findings have implications for public health and clinical practice. In 2024, the National Cancer Institute launched the “Last Mile” Self-collection for HPV Testing to Improve Cervical Cancer Prevention Trial, which aims to establish the accuracy and effectiveness of self-sampled HPV testing in U.S. patients in order to support regulatory approval.^24^ Furthermore, the 2022 President’s Cancer Panel report recommended expanding access to self-sampled tools as a mechanism to increase population-level cancer screening uptake.^25^ Our findings resonate with these national initiatives by contributing to the research base demonstrating the potential impact of self-sampled HPV testing. In particular, our findings demonstrate the acceptability of this testing modality across a wide range of patient characteristics. If self-sampled HPV testing is incorporated into routine clinical practice in the future, it could be an acceptable or preferred option for cervical cancer screening for many patients. In the long term, making this accurate and acceptable tool widely available could reduce disparities in cervical cancer screening and diagnosis.

### Strengths and limitations

Study strengths include analysis of patients with diverse screening histories (i.e., including patients with previous abnormal cervical cancer screening results). In addition, all participants had a recent clinician-collected HPV test, allowing for informed comparisons between the two options for cervical cancer screening. Study limitations include a relatively small sample size (limited by the sample needed for analysis of the primary outcomes, reported elsewhere^14^); future studies should explore acceptability of self-sampled HPV tests with larger samples of average- and high-risk patients. Additionally, the sample demonstrated limited sociodemographic variability because the study took place in the catchment area of a single academic health system in suburban Central Pennsylvania. We also note that some variables related to the acceptability of self-sampled HPV testing had very limited variability (Supplementary Table S1), precluding statistical analysis.

## Conclusions

In conclusion, we demonstrated that self-sampled HPV testing was highly acceptable, a finding that was robust to patients’ level of cervical cancer risk. Altogether, our findings add to existing literature supporting the implementation of self-sampled HPV testing as a routine part of clinical care in the United States, with the ultimate goal of improving cervical cancer screening rates nationally.

## Disclaimer

The content is solely the responsibility of the authors and does not necessarily represent the official views of the National Institutes of Health (NIH).

## Authors' Contribution

R.M.: Conceptualization; methodology; software; formal analysis; investigation; data curation; writing—original draft; writing—review & editing; visualization. A.W.: Conceptualization; validation; investigation; data curation; writing—original draft; writing—review & editing. L.M.W.: Validation; investigation; data curation; writing—original draft; writing - review & editing. A.K.: Investigation; resources; writing—original draft; writing - review & editing. M.M.-M.: Investigation; resources; writing - original draft; writing—review & editing. S.I.R.: Conceptualization; methodology; resources; writing—original draft; writing—review & editing. M.T.R.: Conceptualization; methodology; resources; writing - original draft; writing—review & editing; supervision; funding acquisition. J.L.M.: Conceptualization; methodology; software; formal analysis; investigation; resources; data curation; writing—original draft; writing—review & editing; supervision; project administration; funding acquisition.

## Abbreviation Used

CI: confidence interval
HPV: human papillomavirus
OR: odds ratio
SHIP: Self-collection for HPV testing to Improve cervical cancer Prevention
US: United States
