# Supplementary material for: Motivations and Preferences for Self-Sampled Human Papillomavirus Testing Among Average—and High-Risk Patients: An Exploratory Analysis
Source: Womens Health Rep (New Rochelle). 2025 Mar 28;6(1):377–83. doi: 10.1089/whr.2024.0180 (PMC12040559; doi:10.1089/whr.2024.0180)
Supplement: Supplementary Table S1 [file whr.2024.0180_supp_tables1.docx]

**Supplementary Table S1.** Additional motivating factors for cervical cancer screening and self-sampled human papillomavirus (HPV) testing that demonstrated limited variability in responses among study participants (*n*=46, ages 30-65 years, female sex).

|  | *n* | % |
| --- | --- | --- |
| *Cervical cancer screening facilitators* |  |  |
| Know how to get checked |  |  |
| Strongly/somewhat disagree | 1 | 2% |
| Strongly/somewhat agree | 45 | 98% |
| Have transportation to doctor's office to get checked | |  |
| Strongly/somewhat disagree | 0 | 0% |
| Strongly/somewhat agree | 46 | 100% |
| Partner encourages me to get checked |  |  |
| Strongly/somewhat disagree | 2 | 5% |
| Strongly/somewhat agree | 38 | 95% |
|  |  |  |
| *Sexual history* |  |  |
| New sexual partner in the last month |  |  |
| No | 45 | 98% |
| Yes | 1 | 2% |
|  |  |  |
| *Healthcare factors* |  |  |
| Last-year preventive check-up |  |  |
| No | 4 | 9% |
| Yes | 42 | 91% |
|  |  |  |
| *Feelings during self-sampled test* |  |  |
| Feel embarrassed |  |  |
| Not at all | 46 | 100% |
| Somewhat/very | 0 | 0% |
| Easy to use |  |  |
| False | 1 | 2% |
| True | 45 | 98% |
| Like to do it myself |  |  |
| False | 1 | 2% |
| True | 44 | 98% |
| It was private |  |  |
| False | 0 | 0% |
| True | 46 | 100% |
| It was convenient |  |  |
| False | 2 | 4% |
| True | 44 | 96% |
